# Report Made to the Academy of Sciences in Paris by the Gelatine Commission

**Published:** 1842-04

**Authors:** 


### Art. XV.

1. *Compte Rendu des Séances de l'Académie des Sciences,* No. V.—*Août,* 1841. (*Rapport fait à l'Académie des Sciences au nom de la Commission dite de la Gélatine.*)—*Paris,* 1841. 4to, pp. 77.
*Report made to the Academy of Sciences in Paris by the Gelatine Commission.—Paris,* 1841.
2. *Proceedings of the Philosophical Society of Glasgow.* (*Report of the Physiological Committee "On the best means of providing cheap and nourishing food for the Poor."*)—*Glasgow,* 1842. 8vo, pp. 16.

One of the great obstacles to the discrimination of the nutritive properties of different kinds of food appears to have been a distinction which has been insisted on by many between animal and vegetable matter. Yet if we compare vegetable and animal existence at their beginning, a very close analogy between them must force itself upon even a superficial observer. Both kinds of matter spring from seed; they are nourished by the media which surround them, and in all the relations of mere living beings their conditions are almost parallel. The vegetable kingdom, however, consists of species which are not endowed with the powers of locomotion, and hence we find their food is of a more elementary nature —carbonic acid, ammonia, water; while animals employ substances for their nourishment which are the products of the elaboration of these simpler elements. In this point of view, then, the assimilating apparatus of plants might be considered of a somewhat higher order than those of animals, just in proportion to the greater difficulty in the chemical operation of converting azote, carbon, hydrogen, oxygen, sulphur, and phosphorus into vegetable albumen, than of dissolving that substance in the animal stomach, and depositing it in another part of the body in the solid form of albumen or fibrin, with identically the same chemical composition, and similar or slightly modified physical properties. But further; vegetables are the incipient stage of animal existence, for plants are the only creators of living solids, if we may so speak; they serve to produce, from gases, the solids which, under the term of meat or muscle, we find in cattle in a modified form; so that the human body is purely of vegetable origin. This view has been amply confirmed by chemical analyses, which have demonstrated the identity in composition of the albumen of the pea, the curd of cheese, and the albumen and fibrin of the blood. Henceforth, therefore, it would appear that instead of assuming an animal substance as the type of nutritive food, we must recur to the vegetable kingdom, and form our standard at the source of all organic matter.

These considerations prepare us for appreciating the justice of the views of those who have pointed to the amount of the azote of plants as the indication by which we are to measure the nutritive power of vegetable food. There can be no doubt that an attentive examination of this subject will bear us out in the conclusion that substances derived from the vegetable kingdom are nutritious in proportion to the quantity of azote which they possess. But our present knowledge does not permit us to say that such substances are *alone* endowed with nutritive power. Sugar is perhaps a striking instance in illustration of this exception. The familiar fact of the fattening of the negroes and even dogs during crop-time in the West Indies would appear to negative such a position. Humboldt informs us also that he has frequently observed the mule drivers, who carried his luggage on the coast of Caraccas, giving the preference to unprepared sugar over fresh animal food. In both of these instances, however, it is presumed, unprepared sugar, in other words, the juice of the sugar-cane, which might contain albumen and other matters, was employed; and even these were only used in conjunction with other articles of diet. Potatoes also are universally acknowleged to be far from the bottom of the nutritive scale; and yet the quantity of azote present in this vegetable is exceedingly insignificant compared with the amount which can be detected in even the inferior qualities of wheat. According to Proust, potatoes contain no gluten, nor has any subsequent investigator pointed out accurately the source of the azote which is unequivocally distinguishable in all varieties of this vegetable.

Boussingault, a French chemist, who is well known by his chemical researches in South America, has contributed much to the elucidation of the relative nutritive properties of vegetables. He has examined a variety of the common vegetables employed as human food in connexion with the amount of azote which they contain, and has formed a theoretical table or scale, arranged according to the results of his experiments. He has compared the numbers which he has thus deduced with the practical experience of farmers in feeding cattle, and has detected a most remarkable coincidence between the theoretical and practical inferences. In the following table we have calculated the equivalents, so as to correspond with the vegetables employed in this country. It is read thus: 100 parts of white French beans are equivalent in nutritive power to 120

parts of yellow peas, to 1096 of potatoes, and to 2383 of turnips. A farmer, for example, in feeding cattle, would find that 120 parts of yellow peas would go as far in keeping up the strength and efficacy of his cattle as 2383 parts of turnips; for every one is familiar with the fact that a small portion of oats is sufficient for retaining the strength of a horse unimpaired; while if hay were employed for the same purpose, the amount by weight would require to be much increased:

| | |
|---|---|
| White French beans | 100 |
| Yellow peas | 120 |
| Farina of cabbage | 148 |
| „ of carrots | 170 |
| „ of wheat | 175 |
| Wheat | 191 |
| French Wheat | 193 |
| Rye | 200 |
| Farina of barley | 212 |
| „ of potatoes | 225 |
| Barley | 232 |
| Indian corn | 246 |
| Potatoes | 1096 |
| Carrots | 1351 |
| White cabbage | 1446 |
| Turnips | 2383 |

By an inspection of the preceding table we learn that those vegetables which contain gluten are the most nutritive bodies; while those substances which contain no appreciable quantity of this vegetable principle are comparatively low in the nutritive scale. Gluten is the substance procured from wheat-flour, by digesting the latter in cold water and pressing it through a cloth. By careful washing, the whole of the starch is separated and nothing remains in the cloth but a tough adhesive mass, insoluble in water. To procure this perfectly free from starch is a difficult problem. However, we have found that if it is well washed under a stream of water, and then digested frequently in water, at the temperature of 120°, it may be procured tolerably pure. It should then be digested in alcohol. The latter takes up an oily matter which has been termed *proper gluten*, and leaves vegetable albumen. The quantity of proper gluten is very small, according to our trials. The great bulk, therefore, of what has hitherto been termed gluten is, in reality, albumen consisting of carbon, hydrogen, oxygen, azote, sulphur, and phosphorus, in nearly the same proportions as the casein or curd of milk, the fibrin of the blood, and the albumen of serum and of eggs.

We beg to draw attention to the fact, which has been noted in the preceding observations, viz. that vegetables are nutritive in proportion to the quantity of gluten which they contain; because it has been shown, (Glasgow Report), that in the usual mode of baking bread by fermentation, a portion of this nutritive matter is destroyed in addition to much material which we have already found to be nutritive. In some chemical works, the quantity of gluten contained in flour is stated as high as 24 per cent.; and 18 per cent. is a common quotation. To put this statement to the test of experiment, we procured half a pound of the best Scotch flour, and treated it in the manner previously described. After separating all the starch, we exposed it to the heat of a vapour-bath until it ceased to lose weight. We found the quantity of gluten to amount to

6 per cent. of the original flour. This is a decided proof of the great inferiority of British flour compared with that of Italy and other continental countries. But it is further abundantly proved, by the impossibility of manufacturing British flour into maccaroni, a characteristic property of Italian flour, and which may be viewed as a test of the excellence or deterioration of flour. Those who have examined British maccaroni will at once admit this position; and persons who have been at the expense of preparing it, will still further be enabled to corroborate the accuracy of this statement. It would be very easy to show, if this were the proper place for such discussions, that the corn of this country never can cope in excellence with that grown in milder climates; and our statesmen might hence derive an argument in favour of the unrestricted importation of flour,—from the south, at least,—which could not well be controverted.

In order to form fermented bread, a certain quantity of flour and water is mixed together. Yeast is added to them, and the process of fermentation is allowed to proceed to a certain extent, when it is suddenly stopped by exposure to the powerful temperature of the baker's oven. Now it is necessary, in order to appreciate the nature of this process, that we should follow the yeast in its action. To two pounds of dough, the baker probably adds two teaspoonfuls of yeast. The yeast is a glutinous body, in a state of decomposition, which possesses the power of generating a similar action in sugar, when it comes in contact with that substance. The sugar is decomposed into alcohol and carbonic acid; while the yeast has increased in quantity, from two teaspoonfuls to probably double that quantity. This is obviously proved in the case of the use of leaven, which is quite analogous in its action to yeast. A minute portion of leaven is capable of communicating a decomposing action to a large mass of dough, and a minute part of the dough thus excited to fermentation will occasion a similar action in another mass of dough. It is clear, therefore, that the original mass of ferment has regenerated itself. Now, Liebig, from whom the chemistry of fermentation has received much elucidation, has shown that yeast or ferment, in order to reproduce itself, must act upon gluten; hence it is obvious that in the fermentation of bread, not only the sugar of the flour is acted on, but also the gluten or albuminous and nutritive principle is decomposed. The yeast, moreover, is apt to communicate an impairing flavour to bread, and in this point of view it has been viewed by several scientific men, who have proposed substitutes. Mr. Henry, of Manchester, recommended water impregnated with carbonic acid; and Dr. Colquhoun tried, with various success, to mix alkaline carbonates with acids in the bread, so as to imitate the action of yeast, without imparting to the bread its disagreeable taste. But we are not aware that any one has taken up the subject in an economical point of view, or in relation to the destructive influence of the yeast upon the nutritive matter.

The preceding considerations induced Dr. R. D. Thomson to turn his attention to the subject, and with the assistance of an intelligent baker in the borough (Mr. Dodson), he has been enabled to arrive at some interesting results. Mr. Dodson, who has introduced a highly palatable and very wholesome bread, which is raised by the action of muriatic acid upon carbonate of soda, at his request made an experiment upon a large scale, and found that by the fermenting process, a sack of flour would produce, on an average, a hundred loaves, while from the same quantity of flour, by the use of the acid and alkali, the return was 107 loaves; hence there is a loss of 6½ per cent. by the fermenting process: the deficiency depends upon the conversion of part of the sugar of the flour into alcohol and carbonic acid, also upon the destruction of part of the gluten or albuminous principle, by which means new yeast has been formed. (Glasgow Report). In support of this view, the following reasons are adduced:

According to a mean of eight analyses of wheat from different parts of Europe by Vauquelin, it appears that the quantity of sugar contained in flour amounts to 5·61 per cent. But the quantity lost by baking on the fermented plan, exceeds this number by one per cent. nearly. We are, therefore, necessarily compelled to ascribe this additional loss either to the starch or gluten of the flour. It is difficult to conceive any action which could destroy the starch unless it were first converted into sugar and then fermented; but chemistry does not enable us to suggest such a supposition, while the destruction of the gluten is forced upon us in order to enable us to account for the increase in the quantity of yeast. We conceive that the important fact of the loss of so much sugar and gluten in the production of bread deserves much careful attention. One fifteenth of the whole flour of this country used for the purposes of making fermented bread is blown to the winds. In an economical point of view its importance is paramount. This can be readily demonstrated by an application to figures, and the records of the night asylum for the houseless in one of our provincial towns affords a good illustration. The cost of bread for 1500 persons at that institution is £5 12*s.* 6*d.* If we deduct from this the fifteenth part, the sum will be reduced to £5 5*s.*, or there is a sum of 7*s.* 6*d.* over, which would purchase at 2½*d.* a pound 36 pounds of bread. This would supply 88 additional persons with 6½ ounces of bread, the usual allowance at dinner along with soup; or it would enable most of the 1500 to have a larger share. (Glasgow Report).

With regard to the nutritive power of *sugar,* we have already referred to the condition of the negroes in the West Indies during the season of gathering the sugar crop, when sugar proves nutritive in conjunction with other kinds of food. Most persons are familiar with the experiments of Magendie, in which animals fed on sugar lived well for eight days, and then began to fall off, and ultimately died with all the symptoms of starvation. Starch, as the important constituent of flour, was given to animals by the members of the French Commission, of which Magendie was the reporter, and obviously the main instrument. In the pulverulent form, dogs would not even look at it. When made into a paste, with water, dogs rather than taste it preferred to die of starvation. Even when cooked with butter, lard, sugar, or bread, they refused generally to make use of it; and if some of them did partake of it for a certain time, they never failed to perish of starvation. *Gluten,* however, presented a remarkable contrast to both of the preceding substances. When obtained from wheat-flour or from Indian corn, it presented a phenomenon which did not occur with any of the other immediate organic principles, all of which excite more or less repugnance in the animals to which they were presented. Gluten, whether with a faint odour or with a nauseous smell, was swallowed on the first day without difficulty by dogs, and they continued to make use of it for three months without any interruption. The quantity taken daily amounted to about four or five ounces, and the animals preserved all the characters of perfect health. Gluten, then, appears to present an anomaly; for all the immediate principles, such as fibrin, gelatine, albumen, &c., were never able, when taken *per se*, to contribute to the proper nourishment of animals. Instead, therefore, of depreciating the nutritive power of bread, and impairing its quality by the action of yeast, it would appear that the object ought to be to add more gluten to the flour when that principle is deficient in quantity.

Now, it appears that what was formerly termed *legumin*, or the albuminous principle in peas, exists in this vegetable to a considerable extent. The flour of peas might, therefore, be advantageously used to improve the quality of flour; split peas, ground to a fine powder, would probably be a more grateful addition. By the laboring classes peasemeal is much used, especially in Scotland; and, looking at the physical power of the inhabitants of that country, we should be inclined to conclude that the food which they use must be of a character eminently adapted to sustain strength. By the Scotch labourers, too, fermentation is not considered essential to the production of bread as it is by many who ought to know better. Panification and fermentation are terms which are frequently confounded together. Now nothing can be more obvious than that bread may be either fermented or unfermented, leavened or unleavened. Nor is it necessary for its digestion that bread should be fermented or decomposed, as the operation might be more aptly denominated. The Jew does not labour under indigestion when he has laid aside his leavened bread during the passover, and applied himself to his unleavened cakes. The same observation applies to the barley, peas, and oat-bread of the Scotch peasant, and also to the cakes of Upper India; for we believe that from Delhi to Cabool, the only kind of bread employed is wheat-cakes, similar to what are termed *scones* in Scotland. Biscuits belong to the same category, and are even administered to invalids when other varieties of bread are considered unsusceptible of digestion. Bread, however, as we have seen, may be raised or rendered vesicular by chemical means, without having recourse to the decomposing process; and we doubt not that the means hitherto used for this purpose may be greatly improved, so soon as scientific care shall be bestowed upon this important branch of economy. We have already mentioned a variety of excellent bread baked with carbonate of soda and muriatic acid, now used to a considerable extent in London. We understand also, that Dr. R. D. Thomson, of Glasgow, has succeeded in making an excellent white bread by the employment of ammoniacal alum and sesquicarbonate of soda, which might be particularly recommended to invalids. The former salt consists of 1 atom sulphate of ammonia, 3 atoms sulphate of alumina, and 24 atoms of water. When sesquicarbonate of soda is acted on by this salt, the alumina is precipitated, and sulphate of soda and carbonate of ammonia (which is driven off), are generated, while free carbonic acid is evolved. The consequence is that the bread rises well, even better than that with soda and muriatic acid. The few grains of Glauber salt produced will either have no effect or a beneficial one, and when it is desirable to avoid the formation of even this minute quantity of the salt, instead of sesquicarbonate of soda, the sesquicarbonate of ammonia may be employed. The discharge of gas is slower than when a liquid acid is used; the consequence is that the process bears a more striking resemblance to the common operation of baking by fermentation, and the vesicular structure of the loaf is thus rendered more agreeable. (Glasgow Report.)

We have recommended the addition of peas to flour, on account of the large amount of albumen which the former contains. In the common pea the average quantity is about $18\frac{1}{2}$ per cent.; kidney-beans contain $18\frac{1}{4}$ per cent. The importance of these vegetables in a nutritive point of view is therefore highly worthy of attention. The objections commonly urged to it in consequence of its flatulent nature, may be overcome in a great measure, if not entirely, by digesting it in water at about the temperature of 100°, for several hours before it is used. In soups, for example, it might be used with great advantage to produce a body to that article of diet. Its employment is to be particularly recommended as a wholesome and nutritive ingredient in the food of the poor; and, as will be subsequently shown from the experiments of Magendie, it is greatly to be preferred to the gelatinous matter usually employed in the manufacture of soups. Cabbage, according to Boussingault, is a very nutritive substance, and when dried and reduced to flour it contains a very large quantity of azote.

We have hitherto confined our attention to vegetable food. Experience, however, demonstrates that the diet of man must be presented to him in a variety of forms, and must not be restricted to the vegetable kingdom. At one time it was conceived that scurvy could only be produced by the use of salt provisions. More careful enquiry has, however, shown that scurvy may be engendered by restriction to one kind of diet; so that even vegetable food possesses both a scorbutic and anti-scorbutic agency under particular circumstances. Scurvy frequently attacks the Indian in South America, who lives almost on rice alone. It has reigned epidemically in the rice grounds of Lombardy and Piedmont. The same disease prevailed in an epidemic form in Germany in 1771 and 1772, years of scarcity, when many of the inhabitants were obliged to live on legumes, roots, and even the bark of trees. Scurvy affected numbers of the poor people of France in 1812, 1816, and 1817, when even wild plants were employed as food, in consequence of scarcity. Its effects have been severely felt in India. At the lunatic asylum of Moorshedabad, one third of the inmates are annually affected with scurvy; their diet consists principally of vegetables—all good of their kind. The deleterious influence of a bread and water diet is sufficiently evinced by the fact, that among the prisoners in the gaols of Bengal and Agra, the mortality was 66 per thousand, while among the native troops the deaths were only 10·6 per thousand. How far such treatment of men falling into error is congenial with the benevolent doctrines which "desire not the death of a sinner, but rather that he should turn from his wickedness and live," this is not the place to enquire.

Scurvy is obviously a symptom of bad nutriment—the blood being imperfectly formed or the tissues abnormally deposited. It arises from the want of nourishing food, and a proper admixture of those varieties of diet which were destined for the sustenance of man. The well-known disease at the Millbank Penitentiary—a mixture of scurvy and dysentery—was attributed to the employment of a diet into which succulent vegetables did not enter as an ingredient. Scurvy is therefore one of the forms which starvation assumes, and bears close analogy to the symptoms described by Magendie as occurring to dogs when they were fed upon articles which did not contribute to their nourishment. Starvation in this form has been traced to its true cause after its occurrence for hundreds of years, because it was detected in gaols and madhouses, and was subjected to a careful examination. How many other forms starvation assumes among the poor and helpless is unknown. From the Registrar-General's report for 1839, it appears that 130 persons died of direct starvation—that is, purely from want of food. But how many persons perished of starvation, obscured by other names, has not yet been pointed out by statistical data. Numerous points for enquiry, however, present themselves to the medical statistician in considering this question. How far are typhus, scarlet fever, and other diseases of large towns influenced by bad and imperfect nutriment; and how far does restriction to meager vegetable diet operate upon mortality in our manufacturing towns?

The following table exhibits the mortality of some of our largest towns compared with the deaths over the whole of England and Wales in 1839:

| | | |
|---|---|---|
| Mortality in England and Wales . . . | 2·17 | per cent. |
| Average mortality of towns in England and Wales | 2·62 | " |
| Birmingham . . . . . | 2·58 | " |
| Liverpool . . . . . . | 3·18 | " |
| Glasgow . . . . . . | 3·26 | " |
| Manchester . . . . . . | 3·45 | " |
| Whitechapel . . . . . | 3·86 | " |

From this table we deduce that the mean duration of life in England is 46 years; in towns 38 years; and in Whitechapel 26 years. How far is this mortality attributable to inefficient nourishment? We are convinced that those who have had much intercourse with the poor in their wretched hovels, will bear us out in confirming the propriety of this question. The great burden of the starvation and mortality falls upon the poor; but bringing as it does with it a train of evils and diseases, the consequences must, in some measure, affect the richer portion of the community, in the form of infectious diseases; so true is it that the prosperity of any one class of the population is dependent upon that of all the others.

Experience, the structure of the teeth, and of the digestive organs of the human body, demonstrate that the flesh of animals should enter as an element into the food of man. Some kinds of animal food are digested with much greater rapidity than vegetable food. Bread and coffee require about $4\frac{1}{4}$ hours; fresh beef from 3 to $3\frac{1}{2}$ hours; salt beef from $3\frac{1}{2}$ to $5\frac{1}{2}$ hours; salt pork from $4\frac{1}{2}$ to 6 hours; mutton $3\frac{1}{4}$ to $4\frac{1}{2}$ hours; fowl 4 hours; veal from 4 to $5\frac{1}{2}$ hours; tripe 1 hour; pig's feet 1 hour. But these numbers depend considerably upon the circumstances under which the food is swallowed. If the quantity taken be in excess, slow digestion is the result; and the same remark holds with respect to passions of the mind: exercise also promotes digestion. It is interesting to know that those substances which are most nutritive are not those which are most rapidly digested. Gluten, which, according to Magendie, is exceedingly nutritious, has been found in the stomach five hours after being swallowed. Pig's feet, on the contrary, which are digested in an hour, contain a large proportion of gelatine, which, according to the experiments of Magendie, possesses very inferior nutritive properties. To those who are favoured with the blessings of this life, any of these substances may be procured at will. It is when viewing the conditions of the poor that we must consider diet in an economical point of view; and with them the point is not how to create an appetite, but how to satiate it in the cheapest and most substantial manner.

Papin was the first individual who introduced the method of preparing food from bones, by exposing them to the action of water and steam under pressure in his digester. By this operation a greater quantity of gelatine or animal matter of the bone was dissolved than could be procured by simply boiling bones in water at the ordinary temperature, although Papin considerably exaggerated the advantages of his invention. Papin was a Frenchman, and practised medicine in London, about 1680. He published a work in 1682, on the manner of softening bones, and cooking all sorts of meat; he declared that his digester could transform an old hard cow into excellent meat, (*vache dure et veille en excellente viande.*) At the commencement of the French revolution, when provisions were dear, the attention of men of science was directed to the improvement of the food for the poor. Proust, D'Arcet, and Pelletier took the lead in this attempt at amelioration, but, carried away it appears by the enthusiastic feelings of the times, they made a report to government which future enquiry has not corroborated. The government issued a public instruction to the following effect: "A bone is a tablet of soup formed by nature; a pound of bone gives as much soup as six pounds of meat; bone-soup, in a dietetical point of view, is preferable to meat-soup."—It is obvious, from these high-flown expressions that gelatine and nutritive matter were considered synonymous. Such views continued to be entertained by many Frenchmen, but by none more conspicuously than by the younger D'Arcet, who inherited the gelatine mania from his father. According to him by using the bones of four oxen a fifth is created. At his suggestion the method of extracting gelatine from bones under strong pressure, by the action of steam, was introduced into some of the hospitals of Paris, especially the Hôtel Dieu. The gelatinous solution employed for making the soup at the hospitals, is extracted by submitting to the action of high-pressure steam, the bones proceeding from the meat used in the house; these bones have undergone two decoctions, the first in the morning in preparing the ordinary soup, and the second in the evening for producing the *bouillon maigre.* These bones, after being freed from cartilages, are broken up and are introduced into cast-iron cylinders, where they are exposed to the action of steam, at the temperature of 219° to 221°. The first product of this action is fat, which separates while the gelatinous water cools; the gelatinous fluid is then employed to form the basis of the soups for the sick, being mixed with meat and vegetables. A commission appointed to report upon this soup, gave a favorable opinion of its nutritive power; while the physicians and surgeons of the Hôtel Dieu gave an opposite opinion in the following terms: "The soup made with the gelatinous solution is of bad quality; this soup is more liable to putrefaction than common soup; it possesses a disagreeable taste, which is capable of inducing even disgust; it is less digestible than common soup, and may even impair the functions of the digestive organs; it contains a smaller proportion of nutritive matter than common soup, and the nutritive matter which it does contain, is inferior in quality to that of ordinary soup." The consequence of this report was an order for abolishing the use of the apparatus. But, before removing it, Soubeiran, a distinguished chemist, was consulted; he advised the extraction of the gelatine by means of acids, and disapproved of the vapour method. About the same time, M. Donné made some experiments upon the nutritive properties of gelatine. He swallowed three times a day an ounce and a half of gelatine along with about three ounces of bread. In six days he lost two pounds of weight, and was tormented all the time with the sensation of hunger, and experienced great debility. Two dogs, to which he gave gelatine, refused to partake of it, even when presented to them in an attractive form. M. Gannal, a manufacturer of glue, had observed that rats, which are always ready to devour animal substances, would not touch gelatine nor glue. He made experiments upon five members of his family, and drew the conclusion that gelatine is not only incapable of nourishing human beings, but that it is even hurtful to health, when introduced beyond a certain proportion into regimen. About the same period Edwards and Balzac found that bread and gelatine, when given together, were nutritive but that they are insufficient; that soup and bread are capable of supplying complete nourishment, and that the addition of meat-soup to a regimen of bread and gelatine, is adequate to nourish the human body in a complete manner.

About 1831, a commission was appointed by the French Academy to enquire into the truth of these apparently contradictory statements. The commission, after continuing their experiments for nearly ten years, have now published their report. But the results, so far as their importance is concerned, are neither worthy of the time which has been bestowed upon them nor the space which the report occupies. For with the exception of the observation of the fact, and this we admit is a curious one, that animals can subsist for a considerable period of time on gluten alone, we are not aware that any of the deductions are novel. The first experiments on animal substances were instituted on gelatine. The gelatine was extracted from bones by different methods. But we believe the high-pressure steam system was not adopted. Indeed we think there is every reason to believe that gelatine when acted upon in the manner suggested by D'Arcet, is deprived of ammonia and is converted into glue. So that the soup proposed by D'Arcet might with greater propriety be denominated glue-soup. The gelatine produced in this manner is destitute of the characteristic property of forming a jelly with water. Indeed our surmise is put beyond doubt by the fact stated by those who have examined the soup of the Hôtel Dieu, that the solution of gelatine when evaporated, left a hard extract, presenting the character of Flanders glue. We think it possible, however, that a gelatinous solution might be extracted from bones under pressure, without the disengagement of any ammonia, or without the evolution of any disagreeable odour. We draw this inference from a carefully conducted experiment in which a bone lost by exposure for an hour to a temperature of 230° in a Papin's digester 14½ per cent. The resulting liquid possessed a highly agreeable flavour, nor was the slightest ammoniacal smell perceptible. The advantage of a considerable elevation of temperature is obvious, when we contrast the preceding experiment with another, in which a bone after boiling an hour and a half in water in a common pot lost only 5½ per cent. (Glasgow Report.) Now, according to the French commission, the constitution of bones in an economical point of view is as follows :

| | Sheep's feet bones. | Ox-head bones. |
|---|---|---|
| Water | 47·22 | 22·87 |
| Fat | 5·55 | 11·54 |
| Gelatine | 17·30 | 27·99 |
| Phosphates | 12·42 | 32·77 |
| Insol.-animal matter | 17·51 | 4·83 |
| | 100 | 100 |

From this table we may learn that more gelatine will be extracted by high pressure, when we consider its amount, and when we take into view the facts we have previously adduced.

From the experiments of the commission, we learn no more than we previously knew : viz. that animals are incapable of subsisting upon the exclusive use of an elementary animal principle. The experiments of Donné and Gannal had proved this in reference to gelatine ; but the researches of Edwards had shown, contrary to what the commission seem anxious to admit, that gelatine when administered in conjunction with other articles of diet, may serve for the purposes of nutrition. Gelatine extracted from bones by the action of water is, according to the commission, less nutritive than gelatine extracted by the use of acids. When gelatine prepared by the former method was presented to dogs, some of them partook of it once or twice, while others preferred rather to die than touch it. If the bones were digested in acid, and the residue dissolved in water, dogs lived upon it for a month and were well nourished, especially when the bones were those of sheep's feet: but after this period they exhibited a dislike to this meal. In the choice of acids we find that it is not a matter of indifference which we employ; for by acting upon gelatine by nitric acid, we have procured by distillation by no means inconsiderable traces of prussic acid. Muriatic acid appears to be, considering all circumstances, the most convenient for the object in view. We believe, therefore, that gelatine procured by the action of acids, might be employed advantageously as the basis of soups; but we conceive that the facts previously adduced are sufficiently condemnatory of the process of extracting gelatine from bones by long-continued high-pressure steam.

It would be of essential advantage to the poor, if attention were bestowed on such methods of preparing a cheap and efficient nutriment. We believe that, at least in the present condition of the country, especially among the poor inhabitants of towns, a comfortable meal can rarely be procured ; and that it would be highly advisable to form institutions where poor labourers might be supplied with a cheap meal at their own cost. Under these circumstances it would be necessary to employ cheap materials. But care should be taken that they were of a wholesome and nutritious quality. For this purpose it would be essential that they should be superintended by individuals qualified to judge of the qualities of food, and acquainted with the chemical principles necessarily connected with the various processes employed in preparing and cooking the various articles of diet. We should recommend also that greater variety in the routine of the dietaries should be inculcated. The poor ought not to be fed like the inferior animals, continually upon the same materials. We are convinced, from the proposed improvements in the dieting of the Glasgow poor, that the dinner may be different on each successive day; and that the industrious labourer who constitutes the sinew of the country, may, in his declining years, be treated more in conformity with that class for whose comforts his best energies have been spent, and for whose existence his well-being is essential.

---

